# Sensory stimulation program improves developments of preterm infants in Southwest China: A randomized controlled trial

**DOI:** 10.3389/fpsyg.2022.867529

**Published:** 2022-08-15

**Authors:** Wenjing Zheng, Rassamee Chotipanvithayakul, Thammasin Ingviya, Xiaoling Xia, Lu Xie, Jin Gao

**Affiliations:** ^1^Department of Pediatrics, The Second Affiliated Hospital of Kunming Medical University, Kunming, China; ^2^Department of Epidemiology, Faculty of Medicine, Prince of Songkla University, Hat Yai, Thailand; ^3^Research Center for Kids and Youth Development, Prince of Songkla University, Hat Yai, Thailand; ^4^Department of Family Medicine and Preventive Medicine, Faculty of Medicine, Prince of Songkla University, Hat Yai, Thailand; ^5^Research Center for Applied Medical Data Analytics, Prince of Songkla University, Hat Yai, Thailand; ^6^Department of Neonatology, Kunming University Affiliated Maternal and Child Health Hospital, Kunming, China; ^7^Department of Neonatology, Kunming Children Hospital, Kunming, China

**Keywords:** integrated sensory stimulation, social-emotional development, temperament, growth, neurodevelopment, preterm infant, RCT

## Abstract

Preterm infants are prone to growth and developmental delay, especially social-emotional development. Sensory stimulation may benefit developmental outcomes for these vulnerable infants. This study aims to determine whether 5-integrated sensory stimulation (5-ISS) improves preterm infant social-emotional development. A randomized, parallel trial was conducted from November 2018 to January 2020 at three tertiary hospitals in Kunming, China. Preterm infants were eligible if gestational ages were from 28 to 36 weeks based on ultrasound results when discharged from neonatal wards. Two hundred preterm infants (male *n* = 110, female *n* = 90) were randomly allocated to the 5-ISS intervention group (*n* = 98) and the standard care group (*n* = 102). Social-emotional development was assessed with the Ages and Stages Questionnaires: Social-Emotional (ASQ:SE). Temperament was assessed with the Infant Behavior Questionnaire-Revised. Anthropometry, which included weight, length, and head circumference, was measured at corrected ages of 1, 3, and 6 months. Demographic and clinical characteristics were similar between the intervention and the standard care groups. At 1- and 3-month corrected age, no significant differences between the two groups were observed in terms of infant development and temperament. At 6 months, significant disparities were found in the social-emotional development scale (mean difference −0.29, 95% CI: −0.58, < -0.001, *p* = 0.01), infant length (mean difference 0.70, 95% CI: < 0.001, 1.4, *p* = 0.03), distress to limitation (*p* = 0.04), and sadness (*p* = 0.03). A mixed model revealed that the 5-ISS intervention positively affected social-emotional development, length, distress to limitation, and sadness for preterm infants. Integrated sensory stimulation has benefits on social-emotional development, temperament, and length for preterm infants. This program provides a feasible method to promote social-emotional development for preterm infants.

## Introduction

The estimated preterm delivery rates range globally from 5 to 18% among newborns, which equates to around 15 million cases each year (World Health Organization, 2021). Preterm infants are more vulnerable to poor growth and development than full-term infants due to their immature bodies (Sullivan et al., 2012; Allotey et al., 2018). The brain of a fetus grows rapidly during the third trimester. However, *ex-utero* preterm infants have a smaller brain volume and lower growth trajectory than *in-utero* fetuses (Bouyssi-Kobar et al., 2016). An increase in the preterm infant survival rate due to advances in medical technologies has led to increased morbidity in the short term and ventricular hemorrhage (20–25%), cerebral palsy (10–76%), visual impairment (20%), hearing impairment (5%), and intellectual disability (62.5%) in the long term (Aylward, 2005; Saigal and Doyle, 2008; Back, 2017; Smyrni et al., 2021).

Although some preterm infants do not have obvious physical disabilities, 13–47% have cognitive problems and psychiatric disorders, such as sensorimotor development and less than optimal socio-emotional outcomes (Duncan and Matthews, 2018). Major medical interventions, which are needed to promote the survival rate of preterm infants, often constitute a significant source of stress for infants and families during the NICU stay. These early sources of stress were found to be related to poor socio-emotional developmental outcomes later in life (Malik and Marwaha, 2020). Deprivation of social stimuli in a neonatal intensive care unit (NICU) aggravates infant stress and impairs the learning procedure in processing information and modulating responses. During intensive care, separation from the parents affects maternal mental health, which is one of the most crucial psychosocial factors of infant social-emotional development (Hernandez, 2018). A successful social-emotional process in early life plays a critical role in mental health, achievement in school, and social activities later in life (Thomson et al., 2019).

The developing brain can be modulated within an enriched environment and with pleasurable stimulation (Chorna et al., 2019). The stressful environment of the NICU and early life adversities, such as parental stress, could modify the structure of a developing brain, which is associated with social-emotional impairments (Huhtala et al., 2014; Montagna and Nosarti, 2016; Fumagalli et al., 2018). Social-emotional ability is associated with the right brain that is nurtured by multisensory actions and modulated through a bidirectional interaction (Schore, 2001). During the last few decades, researchers have developed interventions to improve growth and neurodevelopment among preterm infants through many programs such as improving parental mental health, nutrition, massage/skin-to-skin, education on nurturing preterm infants, and mother-infant interaction programs (Feldman et al., 2002; Nordhov et al., 2012; Spittle et al., 2012; Britto and Lye, 2017; Kolb et al., 2017; DeMaster et al., 2019). Previous sensory stimulation programs consisted of one or two sensory stimulations, such as Kangaroo care or skin-to-skin contact, and skin contact plus maternal voice (Conde-Agudelo et al., 2011; Neu et al., 2013; Arnon et al., 2014), and multiple sensory stimulations such as combining all or several sensory organs including auditory, tactile, visual, vestibular, kinesthetic, olfactory and gustatory (VandenBerg, 2007; Vaivre-Douret et al., 2009; Kanagasabai et al., 2013). These programs aimed to improve growth, or neurodevelopment, or both; however, impaired social-emotional development has been overlooked. Moreover, almost all programs were conducted and completed in NICU settings and did not continue when the infants returned home. Therefore, a home-based 5-integrated sensory stimulation (5-ISS) intervention program was designed to maintain the developmental stimulation among preterm infants. The study aimed to assess the effect of the program on growth, temperament, and social-emotional development among preterm infants after 6 months of corrected age.

## Materials and methods

### Study design

A block randomized controlled trial of the 5-ISS intervention program was conducted. It was registered in the Chinese Clinical Trial Registry (no. ChiCTR2000038325). The study procedures were approved by the Institute Ethics Committee of the Faculty of Medicine, Prince of Songkla University and the Medical Ethics Committee in the 2nd Affiliated Hospital of Kunming Medical University. No substantial changes were made to the study design or methods after commencement of the trial.

### Study setting and participants

The study was conducted at three tertiary hospitals including one provincial and two municipal hospitals in Kunming, China from November 2018 to January 2020. Preterm infants were eligible if the gestational age was between 28 and 36 weeks confirmed by ultrasound. Infants were excluded if they presented with traumatic brain injury, severe congenital disease, metabolic or genetic disease, or other serious illnesses and complications. In addition, the dyad was not eligible if the mother was younger than 18 years, had a history of mental illness, substance use, HIV positive, viral hepatitis, syphilis, or other chronic illness, or was unable to care for their baby, or planned to move out of the area during the trial. The required sample size was based on improved social-emotional development by an average improvement of four points [standard deviation (SD) = 1.5] (Spittle et al., 2010) among preterm infants at 6 months corrected age. After considering a 20% attrition rate, at least 100 preterm infants were required in each group, i.e., an intervention group and control group. The type I error was set at 0.05 and power was set at 80%.

### Recruitment and randomization

After NICU discharge, a nurse informed parents about the program and asked if they wanted to participate. Informed consent was obtained from at least one of the parents who agreed to participate in the program. They were asked to complete a questionnaire to obtain socio-demographic information. The histories of the pregnancy and birth were obtained from the hospital records. Infants were randomly assigned to either the 5-ISS or the standard care groups using a block of 4 or 6. The assignment information was placed in an opaque envelope for concealment and the process was done by a person independent from the recruitment of infants and family.

#### Standard care

On the day of discharge, a nurse gave a 30-min general education session and a pamphlet outlining the possible health problems of preterm infants and how to identify abnormal symptoms and deal with them. The parents were instructed on routine care of preterm infants that included encouragement of breastfeeding, amount and frequency of feedings, bathing, cleaning, vaccination schedule, and health screenings along with physical growth and neurodevelopmental milestones. Maternal aspects included the importance of the mother’s role in baby care and to realize and be alert to postpartum anxiety and depression.

#### Interventions

In addition to standard care, the 5-ISS program based on Schore’s Right Brain Dual Corticolimbic-Autonomic Circuits was introduced (Schore, 2001). The program aimed to stimulate social-emotional development through the limbic system of the right brain hierarchically by exteroceptive sensory input. The intervention integrated five sensory systems: (1) Tactile (gentle touch); (2) auditory (voice); (3) visual (eye contact); (4) gustatory (breastmilk, formula milk, or other food); and (5) olfactory (odor of caregiver).

Subsequently, the nurse instructed the use of the five stimulations during feeding time for 10–30 min. The parents were informed to keep the baby’s diaper dry, their own hands dry and warm, and to relax their minds. Other instructions included holding the baby comfortably while feeding, giving a gentle touch (tactile) to any touchable surface, such as the face and hands, if possible, skin-to-skin; facing the baby, and engaging in as much eye contact (visual) with the baby as possible; and speaking (auditory) in their native language about their positive feelings such as love or singing a song such as a lullaby with a smooth rhythm. They were free to start with any type of stimulation, such as speaking with the baby, gentle touch, or eye contact, and the stimulation was to be offered with sensitivity and responsiveness to the infant’s behavioral cues. They could provide the babies with different flavors of milk or various baby food (gustatory) and let the babies smell the original odor of the caregiver (olfactory) by holding the babies close and avoid using perfume. Parents were also asked to watch a 15-min video clip about how to give sensory stimulation to their babies. After the video, the parents were invited to participate in a 30-min hands-on practice session with their own baby under the supervision of a researcher until they could successfully complete the program.

#### Follow-up

The babies were scheduled for monthly follow-ups during the first 6 months after discharge. At each follow-up, pediatricians monitored the growth and development of the babies and ensured that they complied with an immunization schedule. Funduscopic examinations and thyroid function tests were performed as appropriate. The doctor usually prescribed vitamin A and D and gave advice to the caregivers on child-rearing. When the infants were at the 1-, 3-, and 6-month corrected age, a researcher assessed the social-emotional development and neurodevelopment of the babies after completing a routine follow-up assessment.

#### Compliance

After discharge, the parents were asked to conduct the intervention during feeding time each day. In addition they were asked to record in a prepared log the duration of feeding, frequency of feeding, and who conducted the feeding until the infants were 6 months corrected age. Compliance was defined as the application of the intervention for at least 10 min each time and at least three times a day. It was assessed and recorded in the intervention group only.

### Outcomes

At the 1-, 3-, and 6-month corrected age, all preterm infants were assessed for social-emotional development, neurodevelopment, temperament, and anthropological characteristics. All of the psychometric tools are described below.

#### Ages and stages questionnaires: Social-emotional

This is an independent questionnaire to measure infant social-emotional development (Bian et al., 2017; Anunciação et al., 2019). The questionnaire consists of 269 items of social emotional related behaviors that is divided into nine age groups from 1 to 60 months. However, only two age ranges, 1 month 0 day to 2 months 30 days old and 3 months 0 day to 8 months 30 days old, were used in this study. The rating scales consisted of “most of the time” (0 points), “sometimes” (5 points), and “rarely or never” (10 points). Moreover, an additional 5 points should be added if the behavior is of concern to parents. Thus, the maximum score for this item was 10 points. Scores higher than the established cutoff indicated the need for further assessment and/or intervention. McDonald’s Omega was 0.82–0.87 in social and emotional dimension. Cronbach’s alpha ranged from 0.56 to 0.77, and the test-retest reliability was high (0.94).

#### Ages and stages questionnaires

Neurodevelopment for communication, gross motor skills, fine motor skills, problem solving, and personal-social domains were assessed using the ASQ third version. Each domain consists of 6 items (Mei et al., 2015; Alvarez-Nuñez et al., 2020). The rating scales are given 0, 5, and 10 for “No,” “Sometimes,” and “Yes,” respectively. Each domain has its normal reference, i.e., scores below the reference suggest “at risk” of delayed development in that domain. McDonald’s Omega was 0.70–0.81 in subscales. Cronbach’s alpha was 0.8 and the test-retest reliability was also 0.8.

#### Infants behavior questionnaire-revised

Temperament-related behaviors observed over the previous week (or sometimes 2 weeks) were assessed at 3 and 6 months corrected age using the Infants behavior questionnaire-revised (IBQ-R) (Enlow et al., 2016; Wang et al., 2020; Dias et al., 2021). It contained 91 questions categorized into three domains: extraversion; negative affectivity; and orienting/regulation. The rating scales measured frequency of temperament-related behaviors that ranged from 1 (never) to 7 (always). High scores suggested high in extraversion, orientation/regulation, and negative affectivity. McDonald’s Omega ranged from 0.55 to 0.78 in three domains between 3 and 6 months.

#### Anthropometric measures

Growth was assessed by anthropometric measures including weight, length, and head circumference.

### Plan of analysis

All analyses were based on the intention-to-treat principle. The mean (SD) of continuous variables and the frequency and percentage of categorical variables were calculated. Data distribution was examined by the Shapiro test. Changes in scores from pre- to post-intervention were assessed using a paired sample *t*-test. The effect size of the 5-ISS program, compared with the standard care group, was assessed using the Student’s *t*-test at each follow-up. A mixed model was used to estimate the effect of the program over the three follow-up times. The model incorporated fixed effects for each outcome with treatment, time and gestational weeks, and subject-specific random intercepts to account for the correlation between repeated measures. Baseline Residual plots were used to verify linearity. Correlation was used to check independence. A Q-Q plot was used to examine normality of residuals. Bayesian Information Criterion was used to decide the final model. R software version 4.0.5 (CRAN, 2021) and the epicalc (Chongsuvivatwong, 2020), ggplot2 (Wickham, 2016), and lme4 (Bates et al., 2021) packages were used for the data analyses.

## Results

**FIGURE 1:**
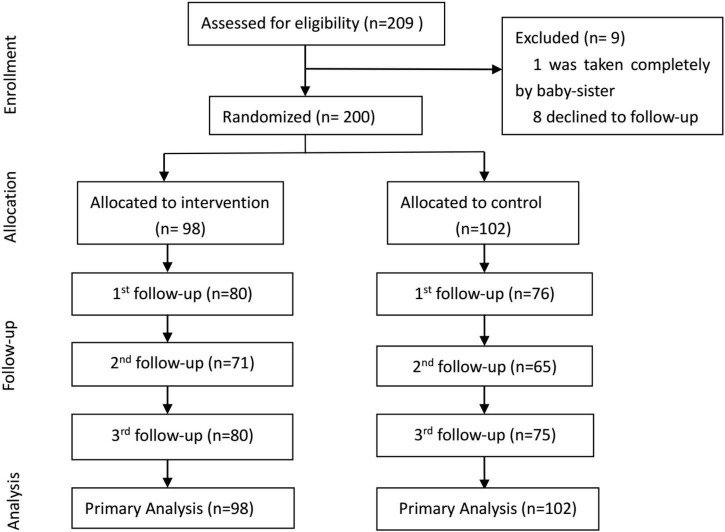
Flow chart of recruitment and follow-up.

A total of 209 preterm infants were consecutively recruited (Figure 1). Nine infants were excluded due to the absence of parents (*n* = 1) and the inability to attend the follow-up appointments (*n* = 8). The remaining 200 infants were randomly allocated to the intervention group (*n* = 98) or the standard care group (*n* = 102). The follow-up attendance rates at 1, 3, and 6 months were 81.6, 72.4, and 81.6% for the intervention group and 74.5, 63.7, and 73.5% for the control group.

### Clinical characteristics of preterm infants

The overall clinical characteristics of the infants in the two groups were similar (Table 1). Approximately one-third of infants were in the early phase of preterm and the remaining were in the late phase. About two-thirds had low birth weight and about a quarter required mechanical ventilation. Infants who developed sepsis or required home oxygen accounted for 2–3% and 5–8%, respectively. The average hospital stay was 2 weeks.

**TABLE 1:** Comparison of infant clinical characteristics between the intervention (*n* = 98) and standard care groups (*n* = 102).

Characteristics	Intervention *n* (%)	Standard care *n* (%)	*P*-value
Gender			0.75
Female	42 (42.9)	48 (47.1)	
Male	56 (57.1)	54 (52.9)	
Gestational age (weeks, mean, SD) Delivery phase	34.5 (1.8)	34.6 (1.8)	0.71 0.85
Early (28–33 weeks)	31 (31.6)	30 (29.4)	
Late (34–36 weeks)	67 (68.4)	72 (70.6)	
Birth weight (grams)			0.42
<2,500	63 (64.3)	72 (70.6)	
2,500–4,000	35 (35.7)	30 (29.4)	
Length at discharge (cm, mean, SD)	45.8 (3.2)	45.6 (2.9)	0.74
Head circumference at discharge (cm, mean, SD)	32.1 (1.8)	31.9 (1.9)	0.37
Delivery mode			0.67
Cesarean	50 (51.0)	48 (47.1)	
Natural	48 (49.0)	54 (52.9)	
Asphyxia (minutes)			
1	16 (16.3)	20 (19.8)	0.65
5	7 (7.1)	4 (4.0)	0.50
10	2 (2.1)	1 (1)	0.62
Mechanical ventilation			
No	68 (76.5)	76 (74.5)	0.82
Without intubation	23 (23.5)	21 (20.6)	0.75
With intubation	7 (7.1)	5 (4.9)	0.71
Sepsis	3 (3.1)	2 (2.0)	0.68
Home oxygen required	5 (5.1)	8 (7.8)	0.62
Length of stay (days, mean, SD)	13.8 (10.7)	14.6 (11.2)	0.43

### Effects of the 5-integrated sensory stimulation on development and growth among preterm infants at 1, 3, and 6 months corrected age

Parents reported acceptable compliance rates of 52, 71, and 66% for the first, second, and third follow-up, respectively.

#### Social-emotional development

**FIGURE 2:**
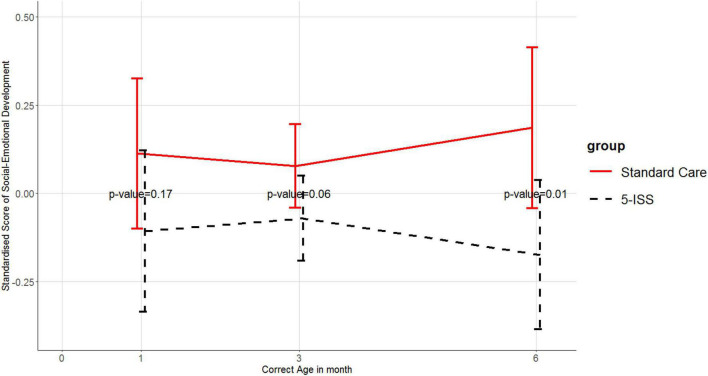
Means and 95% confidence intervals of standardized scores of social-emotional development in the 5-ISS and standard care groups. Higher scores reflect a worse social-emotional outcome.

Assessment of social-emotional development was done after 1 month corrected age as this information was not measurable at baseline. The intervention group had better social-emotional development than the control group but was only statistically significant at the 6-month follow-up (Figure 2).

#### Temperament

Infants in the intervention group had lower negative affectivity scores in subdomains of distress to limitation (mean difference = 0.34, 95% CI: 0.01, 0.68, *p* = 0.04) and sadness (mean difference = 0.35, 95% CI: 0.03, 0.66, *p* = 0.03) than infants in the standard care group at 6 months except for the extraction and orienting/regulation domains (Table 2).

**TABLE 2:** Comparison of temperament subdomain scores at 3 and 6 months between the two groups.

Subdomain	Three months			Six months		
		
	Intervention mean (SD)	Standard care mean (SD)	*P*-value	Intervention mean (SD)	Standard care mean (SD)	*P*-value
Extraction	3.8 (0.9)	3.8 (0.9)	0.87	4.7 (0.8)	4.6 (0.8)	0.41
Activity level	4.0 (1.0)	4.0 (1.0)	0.90	4.8 (0.9)	4.5 (1.0)	0.16
Smiling and laughter	3.8 (1.1)	3.7 (1.0)	0.39	4.5 (1.0)	4.3 (0.9)	0.23
High pleasure	4.5 (1.0)	4.5 (1.1)	0.65	5.4 (0.8)	5.3 (0.9)	0.55
Perceptual sensitivity	3.2 (1.3)	3.1 (1.2)	0.87	3.9 (1.2)	3.9 (1.1)	0.80
Approach	3.7 (1.0)	3.7 (1.2)	0.78	4.7 (0.9)	4.7 (1.1)	0.79
Vocal reactivity	4.0 (1.0)	3.9 (1.2)	0.38	4.8 (1.0)	4.7 (0.9)	0.61
Negative affectivity*	3.5 (0.5)	3.7 (0.5)	0.21	3.7 (0.6)	3.9 (0.6)	0.06
Distress to limitation	3.5 (0.8)	3.8 (0.8)	0.07	3.7 (1.0)	4.0 (1.1)	0.04
Fear	2.7 (0.9)	2.7 (0.9)	1.00	3.0 (1.0)	3.2 (1.0)	0.10
Falling reactivity	4.8 (1.0)	4.8 (0.8)	0.87	5.2 (0.9)	5.0 (0.8)	0.06
Sadness	3.2 (0.8)	3.4 (0.9)	0.15	3.2 (1.0)	3.5 (1.0)	0.03
Orienting/regulation	4.7 (0.6)	4.7 (0.6)	0.83	5.1 (0.6)	4.9 (0.6)	0.15
Duration of orienting	3.3 (1.0)	3.2 (1.1)	0.49	4.1 (1.1)	3.9 (1.1)	0.15
Low pleasure	4.5 (1.0)	4.5 (1.1)	0.65	5.0 (0.9)	4.9 (0.9)	0.57
Soothability	5.4 (0.8)	5.4 (0.8)	0.97	5.5 (0.8)	5.4 (0.9)	0.22
Cuddliness	5.6 (0.7)	5.6 (0.8)	0.59	5.5 (0.8)	5.4 (0.7)	0.27

*Higher scores reflect poorer outcomes.

#### Other neurodevelopment domains

**FIGURE 3:**
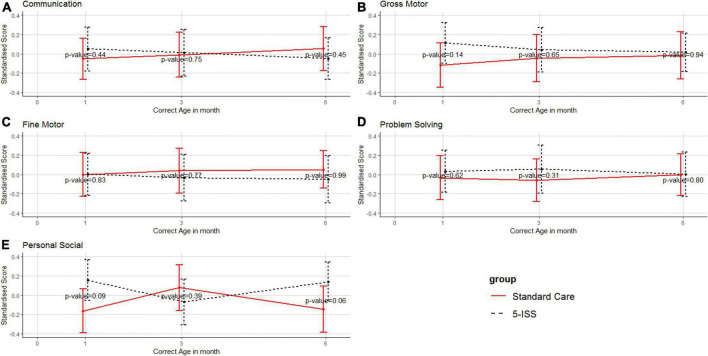
Mean and 95% confidence interval of standardized scores of communication **(A)**, gross motor skills **(B)**, fine motor skills **(C)**, problem solving **(D)**, and personal-social **(E)** between the intervention and standard care groups. Higher scores reflect better neurodevelopmental outcomes.

Other domains, including communication, gross motor skills, fine motor skills, problem solving, and personal-social were stable and no significant differences were observed between the intervention and the control groups (Figure 3).

#### Anthropometry

**FIGURE 4:**
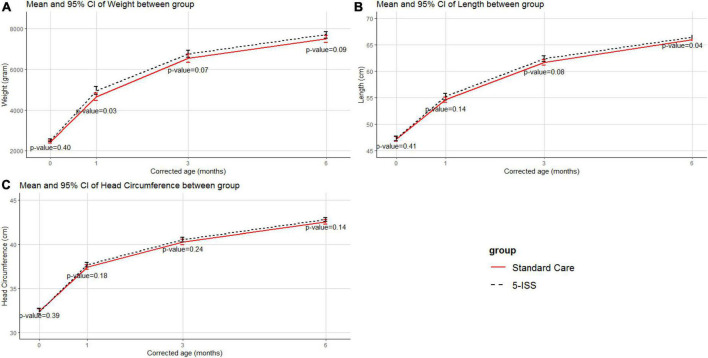
Anthropometry Index between groups in weight **(A)**, length **(B)**, and head circumference **(C)**.

Figure 4 demonstrates similar trajectories for weight, length, and head circumference for the 5-ISS and the standard care groups. Although the 5-ISS group had higher results in all anthropometric measures, a statistically significant result was observed only in length at 6 months corrected age.

#### Effects of intervention on study outcomes using the mixed model

Both time and intervention had a positive effect on infant length but time had a greater effect than the intervention (Table 3). However, the intervention significantly affected only social-emotional development and temperamental negative affectivity that included distress to limitation and behavioral expression of sadness.

**TABLE 3:** Mixed effects model for social-emotional development, length, distress to limitation, and sadness over the 6-month follow-up period.

	Social-emotional development*		Length*		Distress to limitation*		Sadness*	
				
	β Coefficient (95% CI)	*P*-value	β Coefficient (95% CI)	*P*-value	β Coefficient (95% CI)	*P*-value	β Coefficient (95% CI)	*P*-value
(Intercept)	2.67 (0.67, 4.66)	0.01	35.00 (30.41, 39.59)	<0.001	4.99 (2.47, 7.50)	<0.001	3.08 (0.53, 5.65)	0.02
Follow-up (months)	0.01 (−0.07, 0.08)	0.89	6.96 (6.78, 7.13)	<0.001	0.14 (−0.03, 0.31)	0.10	−0.01 (−0.17, 0.14)	0.86
Intervention	−0.27^a^ (−0.48, −0.05)	0.01	0.58 (0.10, 1.07)	0.02	−0.30^a^ (−0.55, −0.04)	0.02	−0.29^a^ (−0.55, −0.02)	0.03

^a^Negative values refer to better outcomes.

*All the models were adjusted by gestational weeks at birth.

## Discussion

Preterm infants are at high risk of delayed social-emotional development due to potential aberrations of brain growth and maturation (Cheong et al., 2017). As exteroceptive multisensory input can nurture the infants’ right brain which is associated with social-emotional development (Schore, 2001), in the present study we explored the effects of a home-based sensory stimulation program, namely 5-ISS. The intervention was performed by caregivers in the home environment. Results highlighted that the 5-ISS could promote preterm infants’ social-emotional development, as well as their temperament and body length at 6 months corrected age.

Our program significantly improved preterm infants’ social-emotional development at a corrected age of 6 months. A plausible explanation of this finding was based on the theory of attachment which indicates that secure attachment has a profound effect on a child’s social-emotional development (Hornor, 2019). In addition, functional magnetic resonance imaging found that amygdala-posterior cingulate connectivity is associated with social-emotional development, but the structure is altered in preterm-born adolescents compared with their full-term peers (Johns et al., 2019). Whereas, mother-infant interaction can promote resonance during brain maturation, and the interaction can regulate emotion-related information in the right brain (Schore, 2001). The 5-ISS program provided an opportunity to increase interaction in the context of eye contact, facial expression, voice, and tactile sensation.

Our study showed that temperament, i.e., an increase in the ability to tolerate distress and sadness, among preterm infants in the intervention group was better than in the control group. Temperament is an innate nature, which links individual differences in behavior and the ability to self-regulate emotional stimulation (Rothbart, 2007). Although temperament is an innate characteristic, it can be modified by environmental factors (Denham et al., 2009). It was shown that affective touch and physical contact represent crucial factors in the development of infants (Mammen et al., 2016). From a physiological perspective, a recent study highlighted that maternal touch buffers the association between the higher methylation level of SLC6A4, which is an important gene in the regulation of the stress-response system, and less than optimal emotional regulation in preterm infants (Mariani Wigley et al., 2021). Multisensory (auditory, tactile, visual, and vestibular) stimulation improved infant responsivity and alert behavior during dyad interaction (White-Traut et al., 2013).

Previous evidence showed some benefits of sensory stimulation on physiology and neuromotor development among infants as well as mother-infant interaction. In line with those results, our study also reported a significant increase in body length at 6 months corrected age. Touching (Feldman et al., 2002; Parsa et al., 2018) or massaging (Diego et al., 2008) was reported to lower heart and respiratory rates with higher temperatures during the intervention along with better mental and psychomotor development and improved mother-infant interaction at 6 months corrected age. When exposed to maternal voice, preterm infants had higher oxygen saturation, more steady autonomic status during and right after the intervention, and had better neuromotor and behavioral skills at 3 months corrected age (Filippa et al., 2013; Picciolini et al., 2014). Even an odor from a sponge soaked with the mother’s amniotic fluid could relieve infant pain during peripheral cannulation (Küçük Alemdar and Güdücü Tüfekci, 2017). Other studies that combined tactile and kinesthetic intervention reported benefits to weight, length, and head circumference after 2 weeks of care (Zhang and Wang, 2019).

Early stimulation by parents at the NICU is ideal to improve development among preterm infants (Spittle and Treyvaud, 2016). However, early stimulation is not always practical in many settings that include infants with a severe illness or visitation restrictions that occur during a global pandemic, such as the COVID-19 outbreak (Murray and Swanson, 2020). Although our program started 2–4 weeks after the infants were discharged from the NICU, the intervention could be performed well at home. This led to improvement in social-emotional development, temperament, and growth of preterm infants at 6 months compared with the standard care group. These findings suggest that the intervention could be started after other physical health problems are resolved, and it takes at least 6 months of the intervention before the benefits emerge.

The strength of our study lies in its randomized controlled trial design, which could control the effect of confounders and minimize biases. Thus, positive findings observed from our study were due to the intervention and not from other factors. The high power of the study was warranted by the adequate sample size and an acceptable follow-up rate over 6 months. In addition, intention-to-treat analysis was done to ensure the benefit from randomization.

However, there are some limitations in our study. First, the study had some risk of contamination, which may have biased the findings toward the null hypothesis since the intervention and control groups were recruited from the same hospitals. Since we could detect a statistically significant effect of the intervention at 6 months, any contamination may be minimal and did not significantly affect the study findings. Second, the study follow-up period was 6 months; therefore, the long-term effects of the intervention are unknown. It is recommended that a long-term follow-up study modified by age and level of development be developed and tested. Third, there was no blinding which could lead to information bias. Last but not least, the data on NICU-related stress experiences and interventions during NICU care were not collected. Consequently, adjustments were not made in the model for those factors that are known to directly affect future outcomes.

## Conclusion

A 6-month home-based 5-ISS program given by a primary caregiver to preterm infants significantly promoted social-emotional development, temperament, and body length. This program provides an opportunity for parents to provide this stimulation program at home post-discharge from the NICU. A study on social-emotional development with a long-term follow-up in a preterm cohort should be conducted.

## Data availability statement

The raw data supporting the conclusions of this article will be made available by the authors, without undue reservation.

## Ethics statement

The studies involving human participants were reviewed and approved by the Human Research Ethics Committee, Faculty of Medicine, Prince of Songkla University. Written informed consent to participate in this study was provided by the participants’ legal guardian/next of kin.

## Author contributions

WZ, RC, and TI contributed to conception and design of the study and performed the statistical analysis. WZ, XX, LX, and JG collected the data and organized the database. WZ wrote the first draft of the manuscript. RC and TI gave comments and revised the whole manuscript. All authors contributed to manuscript revision, read, and approved the submitted version.
